# Dynamic Bayesian network modeling for intervention mechanism

**DOI:** 10.1186/1471-2202-13-S1-P24

**Published:** 2012-07-16

**Authors:** Yan Sun, Yi-Yuan Tang

**Affiliations:** 1 Research Center of Psychological Development and Education, Liaoning Normal University, Dalian 116029, China; 2 Psychology Department of Education School, Liaoning Normal University, Dalian 116029, China; 3 Texas Tech Neuroimaging Institute and Department of Psychology, Texas Tech University, Lubbock, TX79409, USA; 4 Institute of Neuroinformatics, Dalian University of Technology, Dalian 116024, China

## 

Functional magnetic resonance imaging (fMRI) is an important experimental tool in neuroscience. However, how to analyze the fMRI data effectively and accurately has become one of the major challenges in computational neuroscience [1]. Integrative body-mind training (IBMT) was adopted from traditional Chinese medicine and has been proven to improve attention and self-regulation compared to same amount of relaxation training using regular neuroimaging analysis methods in our previous studies [2,3]. The greatest advantage of dynamic Bayesian networks (DBNs) is that it could demonstrate the temporal and causal relationships among different brain regions more accurately. We here propose DBNs to identify the brain changes using the fMRI data sets of five days of IBMT intervention. At first, we employed Statistical Parametric Mapping software (SPM8, http://www.fil.ion.ucl.ac.uk/spm) to preprocess the images. Second, the Markov chain was introduced to model the fMRI time-series and obtained the temporal relationships among brain regions. Third, we used K2 algorithm to learn the structure of the DBNs and adopted the greedy search algorithm to search for the local best optimal connectivity structure from fMRI data [4]. Finally, we obtained the DBNs of the IBMT group and relaxation group, which represent the interactions among brain regions with temporal processes. The nodes in the DBN represented the activations of brain regions at a specific time while the edges denoted the connectivity strengths between brain regions. The DBNs of IBMT group was different from that of relaxation group in the several brain regions particularly in the anterior cingulated cortex (ACC), which was consistent with our previous research findings [3]. The DBNs is an efficient method to demonstrate the brain mechanism of short-term meditation intervention.
